# Association between body mass index and anti-Müllerian hormone in women with ovarian endometrioma and dermoid cyst

**DOI:** 10.3389/fendo.2026.1746451

**Published:** 2026-02-19

**Authors:** Yunjeong Park, Hyemin Park, Inha Lee, Jae Hoon Lee, SiHyun Cho, Young Sik Choi

**Affiliations:** 1 Institute of Women’s Life Medical Science, Yonsei University College of Medicine, Seoul, Republic of Korea; 2 Department of Obstetrics and Gynecology, Severance Hospital, Yonsei University College of Medicine, Seoul, Republic of Korea; 3 Department of Obstetrics and Gynecology, Gangnam Severance Hospital, Yonsei University College of Medicine, Seoul, Republic of Korea

**Keywords:** anti-Müllerian hormone, body mass index, dermoid cyst, ovarian endometrioma, ovarian reserve

## Abstract

**Background:**

Adiposity influences reproductive function via endocrine and immune pathways. The association between body mass index (BMI) and anti−Müllerian hormone (AMH) in endometriosis is uncertain, and BMI may not fully capture adiposity−related biology relevant to ovarian reserve. We assessed whether BMI is associated with AMH in untreated ovarian endometrioma and whether this differs from dermoid cysts.

**Methods:**

Retrospective single−center cohort of 951 newly diagnosed, reproductive−age women from January 1, 2020 to December 31, 2023 (717 endometrioma; 234 dermoid). AMH was measured on one platform; imaging included transvaginal ultrasonography with MRI or contrast−enhanced abdominopelvic CT as needed. Multivariable linear regression modeled log−AMH versus BMI, adjusting for age, diagnosis, cyst size and laterality, parity, smoking, alcohol use, cycle regularity, and cycle length. Nonlinearity was screened with restricted cubic splines; piecewise models explored age breakpoints. An interaction term tested whether the BMI effect differed by diagnosis. Effects are reported as percent change in AMH per 1 kg/m².

**Results:**

Women with endometrioma were older (31.9 vs 29.9 years; P<.001) and had lower BMI (21.1 vs 22.4 kg/m²; P<.001) than those with dermoid. Median AMH was 2.52 vs 2.70 ng/mL; age−adjusted geometric means did not differ (P = .245). Piecewise modeling identified earlier age breakpoints in endometrioma (35.7 years) than dermoid (40.4 years). In fully adjusted models, each 1 kg/m² higher BMI was associated with 2.3% lower AMH (P = .003). Group−specific estimates were −1.9% per kg/m² in endometrioma (P = .060) and −2.8% per kg/m² in dermoid (P = .009); the BMI×diagnosis interaction was not significant (P = .538). Model fit was modest (adjusted R²=0.22), and BMI explained 1% of AMH variance (partial R²=0.01). Sensitivity analyses restricting the BMI range yielded consistent directions of effect with attenuation at lower BMI.

**Conclusions:**

Across endometrioma and dermoid cysts, BMI shows a weak inverse association with AMH without evidence of between−group differences. Given BMI’s minimal explanatory value, local ovarian factors may more strongly determine ovarian reserve in endometrioma. Limited numbers of obese participants constrain inference at higher BMI; studies with broader BMI distributions and integrated metabolic profiling are warranted.

## Introduction

Adiposity plays an important role in female reproductive health. Adipose tissue functions not only as an energy reservoir but also as an active endocrine and immune organ, influencing the hypothalamic–pituitary–ovarian (HPO) axis and modulating inflammatory responses (1). Deviations of BMI from the normal range can disrupt reproductive function and result in menstrual disturbances (2).

Because BMI does not fully capture adiposity, fat distribution, and adipose−immune activity (3), it may obscure mechanistic links between metabolic state and ovarian function (4). This is particularly relevant to endometriosis, a chronic inflammatory condition in which many women maintain relatively regular menstrual cycles despite appreciable effects on ovarian reserve (5).

Epidemiological data indicate that affected women tend to have a lower BMI than unaffected women. However, BMI does not consistently correlate with disease severity; measures such as ovarian endometrioma size, symptom burden, and laparoscopic staging often show poor concordance (6).

Although AMH is widely used as an indicator of ovarian reserve (7, 8), evidence regarding its association with BMI in endometriosis remains to be elucidated. Some studies report more severe disease in obese women with endometriosis, whereas serum AMH is often reduced in advanced endometriosis, particularly in the presence of ovarian endometrioma (9, 10). Taken together, these observations point to a complex, potentially non−linear interplay between adiposity and ovarian reserve. Given that AMH levels may help elucidate the interplay between adiposity, ovarian function, and endometriosis severity, further investigation is warranted.

Therefore, we aimed to quantify the association between pre−treatment BMI and serum AMH in women newly diagnosed with ovarian endometrioma, before any medical or surgical intervention. By clarifying this relationship, our findings may enhance pre−treatment counseling (e.g., expectations for ovarian reserve and timing of fertility preservation) and inform individualized management in endometriosis.

## Materials and methods

### Study design and population

This retrospective cohort study was conducted at a single tertiary referral center. Patient data were retrieved using the Severance Clinical Research Analysis Portal (SCRAP), an institutional electronic data warehouse that integrates clinical, laboratory, and administrative data from electronic medical records. We identified women who were newly diagnosed with ovarian endometrioma and presented to Severance Hospital between January 1, 2020 and December 31, 2023. In addition, a comparator cohort of women who were newly diagnosed with ovarian dermoid cyst and pathologically confirmed after surgery during the same period was identified from SCRAP.

### Inclusion criteria

Eligible patients were (1): women aged 20–45 years (2); newly diagnosed with ovarian endometrioma or ovarian dermoid cyst between January 1, 2020 and December 31, 2023 (3); not previously treated for endometriosis (surgery, medication, or sclerotherapy) or dermoid cyst (surgery); and (4) had serum AMH measured at the initial visit.

Dermoid cysts were selected as a pragmatic comparator because they share the clinical evaluation pathway of an adnexal mass but are not defined by the estrogen−dependent chronic inflammatory process that characterizes endometriosis (5), and have been used as a clinical comparator in prior studies assessing preoperative AMH in women with ovarian cysts (10). Because dermoid cysts were restricted to surgically treated and pathologically confirmed cases to minimize misclassification, the dermoid cohort should be interpreted as a surgically selected ovarian−cyst comparator rather than a population−based ‘healthy control’ group.

### Exclusion criteria

Patients were excluded if they had: missing AMH data or AMH ≤0.01 ng/mL; missing essential variables (height, weight, age, cyst size); prior treatment or recurrent disease; recent (<3 months) hormonal treatment, pregnancy, lactation, or ovarian stimulation; polycystic ovary syndrome, premature ovarian insufficiency, or early menopause; uncontrolled endocrine disorders; prior hysterectomy or adnexal surgery; history of malignancy, autoimmune disease, or severe hepatic/renal disease; or exposure to gonadotoxic drugs, chemotherapy, or pelvic radiotherapy. Because polycystic ovary syndrome and major endocrine/metabolic disorders can substantially affect AMH levels and are closely associated with BMI, they can introduce major confounding in studies of BMI–AMH associations. We therefore excluded these conditions *a priori* to improve interpretability of the estimated BMI effect. Patients with concomitant endometriosis were excluded from the comparator group. Likewise, patients in the endometriosis group were excluded if other types of ovarian cysts were present.

### Clinical variables

Demographic, anthropometric, and lifestyle data were obtained from EMRs. BMI (kg/m²) was calculated from measured height and weight, and analyzed both as a continuous variable and as a categorical variable according to the World Health Organization (WHO) Asian classification: underweight (<18.5), normal (18.5–22.9), overweight (23.0–24.9), and obese (≥25.0) Ovarian cyst size was defined as the sum of the maximum diameters of the right and left ovarian cysts. Smoking status (never vs ever), alcohol consumption (five categories by weekly frequency), and menstrual cycle characteristics (regular vs irregular; self-reported cycle length for regular cycles) were included as covariates.

### Outcomes

The primary outcome was the association between BMI and serum AMH levels in women with ovarian endometrioma, with comparisons to a dermoid cyst control group (comparator). Secondary analyses assessed AMH differences across BMI and age subgroups, and evaluated the influence of potential covariates (cyst laterality, cyst size, menstrual cycle characteristics, parity, smoking, and alcohol use). Exploratory analyses tested for BMI×group interactions to determine whether the BMI–AMH relationship differed between endometrioma and dermoid cyst group.

### Laboratory and imaging methods

Serum AMH concentrations were measured using the cobas e801 modules (Roche Diagnostics International AG; Rotkreuz, Switzerland) according to the manufacturer’s instructions. The intra- and inter-assay coefficients of variation were <5%. For adnexal mass evaluation, all patients underwent transvaginal ultrasonography; if the diagnosis was inconclusive, additional MRI or contrast-enhanced abdominopelvic CT was performed. Ovarian cyst size was defined as the sum of the maximum diameters of right and left ovarian cysts, measured on transvaginal or transrectal ultrasonography by obstetrician−gynecologists or dedicated sonographers in the Department of Obstetrics and Gynecology, and on MRI/CT (when performed) based on radiology reports interpreted by board−certified radiologists. Because imaging was obtained as part of routine clinical care, interpreters were not formally blinded to clinical information; serum AMH results were available in the electronic medical record and could be accessed if needed.

### Statistical analysis

The primary analysis focused on the association between BMI and log−AMH within the endometrioma group. *A priori*, we powered the study to detect a small incremental association of BMI with log−AMH, operationalized as a partial R² =0.01–0.02 (two−sided α=0.05), consistent with published effect sizes of roughly 8–12% lower AMH per 5 kg/m² higher BMI (11). Accordingly, we estimated that 700 endometrioma participants would provide 80% power (12). Dermoid participants were enrolled as a comparator to enable secondary/exploratory assessment of effect modification (BMI×diagnosis); subgroup/interaction analyses were not formally powered.

Baseline characteristics were compared using t−tests or Mann–Whitney U tests, and χ² or Fisher’s exact tests for categorical variables; standardized mean differences (SMDs) were reported. For AMH between groups, analysis of covariance (ANCOVA) was performed on log−AMH with age as a covariate and results back−transformed to percent differences with 95% confidence intervals (CIs).

To characterize age–AMH trajectories, we used segmented (piecewise) regression by group to estimate data−driven breakpoints and compared pre−/post−break slopes.

We regressed log−transformed AMH on BMI (continuous) using multivariable linear models, adjusting for age, diagnosis, ovarian cyst maximum diameter, bilaterality, parity, smoking, alcohol use, cycle regularity, and cycle length. Age, cycle length, and cyst size were specified with restricted cubic splines (RCS, df=4). Cyst size and laterality were included *a priori* to partially account for potential cyst−related effects on AMH across cyst phenotypes (10). BMI non−linearity was screened in age−adjusted models via nested F−tests comparing linear vs spline terms; BMI was kept linear in the primary specification. Spline-based curves were used for model diagnostics and visualization, and any inference about the shape of the BMI–AMH relationship (e.g., attenuation across BMI ranges) was treated as exploratory. A group × BMI interaction tested differential effects by diagnosis. Coefficients on the log scale were exponentiated to express percent change in AMH per 1 kg/m² increase in BMI.

Pre−specified secondary analyses included (i) age−stratified BMI–AMH associations using breakpoints from segmented models with formal BMI × age−group tests; (ii) categorical BMI analyses (WHO categories) with age−adjusted geometric means and group × category interactions (Dunnett adjustment); and (iii) BMI−range−restricted sensitivity analyses (≤30, and ≤35 kg/m²) to assess robustness and potential range restriction; interpretation of differences across BMI ranges was considered exploratory. Model assumptions were checked with standard diagnostics.

All tests were two−sided with α = 0.05. Analyses and figure generation were performed using R version 4.5.1 (R Foundation for Statistical Computing, Vienna, Austria).

### Ethics statement

This study was conducted in accordance with the principles of the Declaration of Helsinki and was approved by the Institutional Review Board of Severance Hospital (IRB No. 4-2025-1027). The requirement for informed consent was waived owing to the retrospective nature of the study.

## Results

### Baseline characteristics

A total of 951 women were included: 717 with endometriomas and 234 with dermoid cysts (**Supplementary Figure S1**; see Additional file). Baseline characteristics differed between groups (**Table 1**; see end of document). Women with endometriomas were older (31.9 ± 6.1 vs 29.9 ± 5.9 years, p < 0.001) and had lower BMI (21.1 ± 3.0 vs 22.4 ± 4.1 kg/m², p < 0.001). Menstrual cycles were more often regular (92.1% vs 78.9%, p < 0.001) and shorter (28.8 vs 30.6 days, p < 0.001) in the endometrioma group. Median serum AMH was 2.52 ng/mL in the endometrioma group and 2.70 ng/mL in the dermoid group (p = 0.024 by rank-sum), but age-adjusted geometric means did not differ (2.18 vs 2.34 ng/mL; p = 0.245; **Supplementary Table S1**; see Additional file).

**Table 1:** Baseline characteristics of the study population.

Variable	Endometrioma (n=717)	Dermoid (n=234)	SMD	p-value
Age, years	31 [28–36]; 31.9 ± 6.1	29 [26–34]; 29.9 ± 5.9	0.334	<0.001
Height, cm	163.0 [159.7–166.2]; 162.8 ± 5.1	162.0 [158.3–166.0]; 162.4 ± 5.3	0.083	0.131/0.267
Weight, kg	55.0 [50.1–60.0]; 56.0 ± 8.4	56.9 [51.8–63.4]; 59.2 ± 11.7	0.315	0.001/<0.001
BMI, kg/m²	20.6 [19.1–22.7]; 21.1 ± 3.0	21.4 [19.7–23.9]; 22.4 ± 4.1	0.361	<0.001
BSA, m²	1.58 [1.51–1.65]; 1.59 ± 0.12	1.61 [1.52–1.68]; 1.62 ± 0.15	0.247	0.016/<0.001
AMH, ng/mL	2.52 [1.36–4.07]; 3.0 ± 2.3	2.70 [1.81–4.34]; 3.3 ± 2.4	0.149	0.024/0.046
CA-125, U/mL	41.0 [25.2–70.8]; 61.3 ± 69.7	17.1 [12.4–25.7]; 24.3 ± 24.4	0.708	<0.001
Dysmenorrhea (VAS)	6.5 [4.5–8.0]; 6.0 ± 2.5	4.5 [2.5–6.5]; 4.0 ± 2.6	0.806	<0.001
Cyst size, max, cm	5.0 [3.8–6.5]; 5.4 ± 2.5	6.2 [4.5–8.3]; 7.2 ± 4.2	0.513	<0.001
Cyst bilaterality, n (%)	No 499 (69.6) Yes 218 (30.4)	No 187 (79.9) Yes 47 (20.1)	0.239	0.002
Cycle regularity, n (%)	Regular 641 (92.1) Irregular 55 (7.9)	Regular 180 (78.9) Irregular 48 (21.1)	0.380	<0.001
Cycle length, days	28 [28–30]; 28.8 ± 3.5	30 [28–30.5]; 30.6 ± 5.6	0.396	<0.001
Parity, n (%)	0: 623 (86.9) 1: 50 (7.0) 2: 42 (5.9) ≥3: 2 (0.3)	0: 195 (83.3) 1: 24 (10.3) 2: 14 (6.0) ≥3: 1 (0.4)	0.121	0.336
Alcohol, n (%)	None: 373 (56.3) Occasional (<1/month): 23 (3.5) Monthly (1–3/month): 105 (15.9) Weekly (1–3/week): 155 (23.4) Frequent (≥4/week): 6 (0.9)	None: 114 (56.7) Occasional (<1/month): 23 (11.4) Monthly (1–3/month): 32 (15.9) Weekly (1–3/week): 31 (15.4) Frequent (≥4/week): 1 (0.5)	0.352	<0.001
Smoking, n (%)	No 662 (92.5) Yes 54 (7.5)	No 201 (90.5) Yes 21 (9.5)	0.069	0.395

AMH, anti-Müllerian hormone; BMI, body mass index; BSA, body surface area; CA-125, cancer antigen-125; VAS, visual analogue scale; SMD, standardized mean difference. Continuous variables are shown as median [IQR] and mean ± SD, and categorical variables as n (%). Standardized mean differences (SMDs) between groups are provided.

### Age and AMH association

In both groups, AMH declined significantly with increasing age (**Supplementary Figure S2**; see Additional file). Segmented regression identified breakpoints at 35.7 years (endometrioma group) and 40.4 years (dermoid group) (**Supplementary Table S2**; see Additional file). The post-break decline was steeper in the dermoid group (β = −0.51 ± 0.15) than in the endometrioma group (β = −0.21 ± 0.02), with a between-group difference reaching significance (p = 0.047), whereas pre-break slopes did not differ (p = 0.790).

### BMI and AMH association

**Figure 1:**
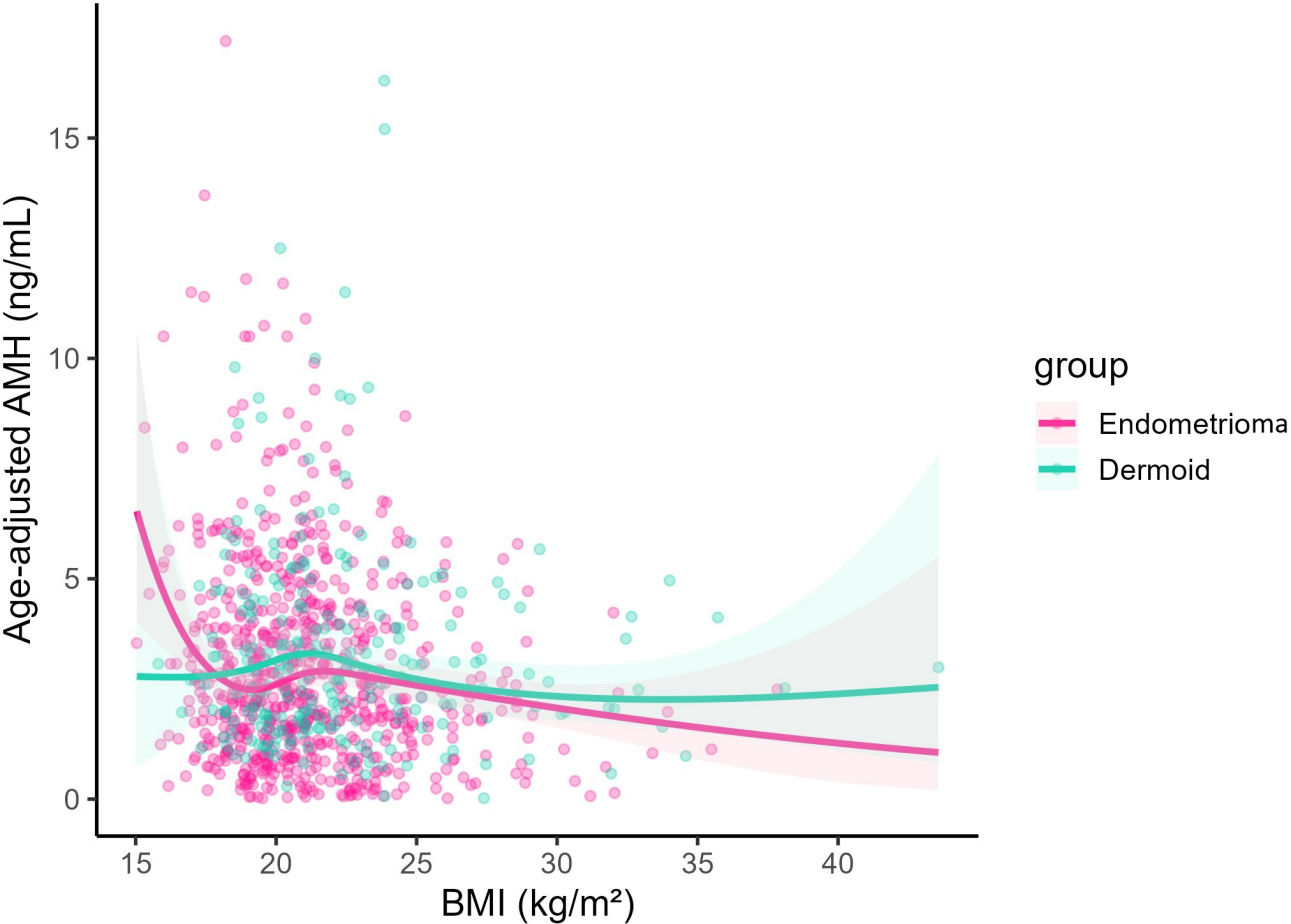
Association between BMI and serum AMH after age adjustment using restricted cubic splines (RCS, df = 4). Curves represent predicted AMH values at the median age, with shaded areas indicating 95% confidence intervals. Curves depict model−predicted AMH values (not raw observations) from the age−adjusted restricted cubic spline model. Separate curves are displayed for women with endometrioma (pink) and dermoid cyst (mint). The y-axis is shown on the logarithmic scale (values back-transformed to ng/mL).

After age adjustment using restricted cubic splines (RCS), higher BMI was associated with lower AMH (**Figure 1**). The RCS model fit the data better than a linear specification (F = 4.08, p = 0.007; **Supplementary Table S3**; see Additional file), indicating modest nonlinearity, but the overall trend remained inverse. In multivariable models (**Table 2**; see end of document), age was the strongest determinant (per 9 years: −38.9%, p < 0.001 overall), and women with endometriomas had lower AMH than those with dermoid cysts (−15.8%, 95% CI −24.9% to −5.7%; p = 0.002). For BMI, each 1−kg/m² increase was associated with a 2.3% decrease in AMH overall (p = 0.003). For interpretability, this corresponds to an estimated 11% lower AMH per 5 kg/m² higher BMI, conditional on covariates. In group-specific models, the inverse BMI–AMH association was significant in the dermoid group (β = −0.03; −2.8% per kg/m²; p = 0.009) but not in the endometrioma group (β = −0.02; −1.9% per kg/m²; p = 0.060). However, the BMI × group interaction was not significant (Model 5 interaction p = 0.538; **Supplementary Table S4**; see Additional file), indicating no evidence of a differential BMI effect between groups. Overall model fit was modest (adjusted R² = 0.22). BMI provided minimal incremental explanatory value after covariate adjustment (partial R² = 0.01), accounting for 1% of the remaining (residual) variance in log−AMH (**Supplementary Table S5**; Additional file).

**Table 2:** Multivariable regression of serum AMH on age, group, and BMI.

Predictor	Contrast/function	β (Adj)	% Change (Adj)	P-value (Adj)	β (Endo)	% Change (Endo)	P-value (Endo)	β (Dermoid)	% Change (Dermoid)	P-value (Dermoid)
Age	per 9 yrs (27→36)	-0.493	-38.9%	<0.001	-0.504	-39.6%	<0.001	-0.502	-39.5%	<0.001
Group	Endometrioma vs Dermoid	-0.172	-15.8% (-24.9 to -5.7%)	0.002	—	—	—	—	—	—
BMI	per 1 kg/m²	-0.023	-2.3% (-3.7 to -0.8%)	0.003	-0.019	-1.9% (-3.8 to 0.1%)	0.060	-0.029	-2.8% (-4.9 to -0.7%)	0.009
Cyst size	Max (cm, RCS)	—	—	0.029	—	—	0.144	—	—	0.293
Bilateral	Yes vs No	-0.107	-10.1% (-19.5 to 0.4%)	0.060	-0.101	-9.6% (-20.5 to 2.7%)	0.120	-0.115	-10.9% (-28.9 to 11.7%)	0.310
Parity	1 vs 0	0.027	2.7% (-15.9 to 25.4%)	0.770	-0.007	-0.7% (-22.7 to 27.6%)	0.960	0.099	10.4% (-20.4 to 53.0%)	0.520
	2 vs 0	-0.146	-13.6% (-32.0 to 9.8%)	0.240	-0.168	-15.5% (-36.4 to 12.3%)	0.260	-0.179	-16.4% (-46.4 to 30.3%)	0.380
	≥3 vs 0	-0.580	-44.0% (-77.8 to 41.0%)	0.180	0.127	13.5% (-64.2 to 260.1%)	0.850	-2.519	-91.9% (-98.2 to -63.0%)	<0.001
Alcohol	1 vs 0	-0.161	-14.9% (-32.7 to 7.8%)	0.190	-0.132	-12.3% (-37.7 to 23.4%)	0.500	-0.194	-17.7% (-40.2 to 13.3%)	0.250
	2 vs 0	0.001	0.1% (-13.6 to 16.0%)	0.980	0.015	1.5% (-14.8 to 21.0%)	0.870	-0.045	-4.4% (-27.3 to 25.6%)	0.760
	3 vs 0	-0.077	-7.4% (-18.8 to 5.6%)	0.270	-0.069	-6.7% (-19.8 to 8.6%)	0.380	-0.122	-11.5% (-32.6 to 16.2%)	0.400
	4 vs 0	0.420	52.2% (-12.3 to 164.2%)	0.120	0.437	54.8% (-15.5 to 183.8%)	0.130	0.374	45.3% (-66.4 to 529.0%)	0.500
Smoking	Yes vs No	-0.212	-19.1% (-32.7 to -2.8%)	0.023	-0.222	-19.9% (-35.9 to 0.1%)	0.051	-0.176	-16.1% (-39.5 to 16.3%)	0.300
Cycle reg.	Irregular vs Regular	0.237	26.8% (9.8 to 46.4%)	0.002	0.190	21.0% (-1.1 to 47.9%)	0.064	0.303	35.3% (11.1 to 64.8%)	0.002
Cycle length	Non-linear function	—	—	<0.001	—	—	<0.001	—	—	<0.001

Log-transformed AMH was modeled using restricted cubic spline (RCS) functions (df = 4). Regression coefficients (β) are shown on the log scale with 95% confidence intervals (CIs), along with the corresponding percent changes after back-transformation. The “Adj” columns indicate the combined model adjusted for age and group, whereas “Endo” and “Dermoid” columns present group-specific models. Non-linear terms are reported for cyst size and menstrual cycle length, and categorical variables are presented relative to the reference category.

% change (Percent change) was calculated as [exp(β) − 1] × 100.

AMH, anti-Müllerian hormone; BMI, body mass index; RCS, restricted cubic spline; CI, confidence interval.

### Secondary and supporting analyses

**Figure 2:**
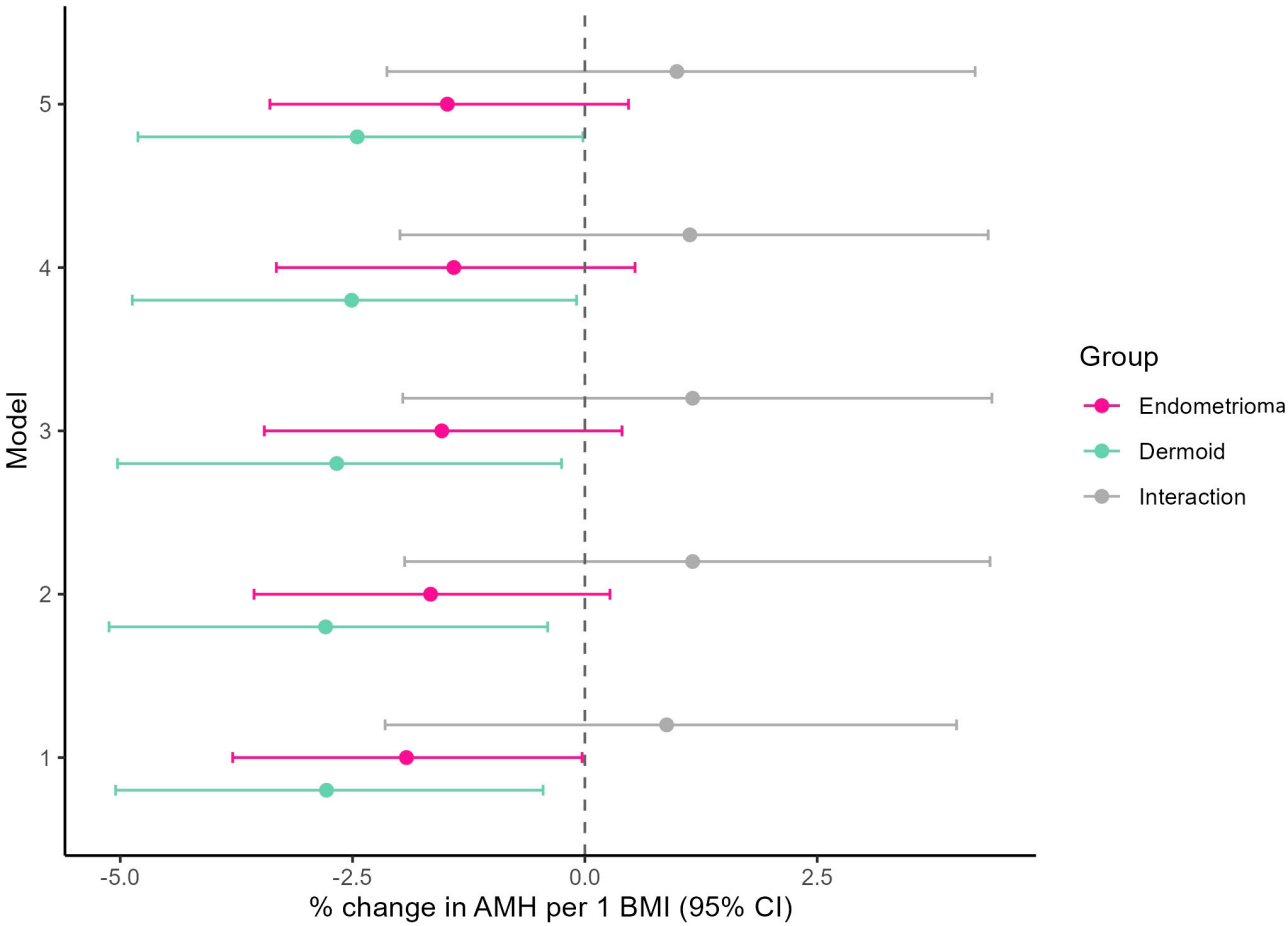
Forest-plot–style graphical presentation of the association between BMI and AMH corresponding to the sequential multivariable regression models in **Supplementary Table S4**. Each point represents the percent change in AMH per 1 kg/m² increase in BMI, with horizontal bars indicating 95% confidence intervals. Results are shown separately for women with endometrioma (pink) and dermoid cyst (mint), with gray markers denoting the interaction terms that represent the differential BMI effect between groups. All points and confidence intervals reflect model−derived estimates from sequential regression models. Models were adjusted stepwise: Model 1, age; Model 2, + cycle regularity; Model 3, + smoking; Model 4, + maximum cyst diameter; Model 5, + parity.

Across stepwise models (Models 1–5), the inverse BMI effect persisted in direction in both groups (**Supplementary Table S4**; **Figure 2**; see Additional file). In Model 1 (age-adjusted), the association per 1 kg/m² was −1.9% (p = 0.047) in the endometrioma group and −2.8% (p = 0.020) in the dermoid group; in Model 5 (fully adjusted), estimates were −1.5% (p = 0.135) and −2.5% (p = 0.048), respectively, while interactions remained non−significant (p = 0.468–0.573). Model fit and incremental variance explained are summarized in **Supplementary Table S5** (Additional file).

When stratifying by age breakpoints, BMI remained inversely related to AMH within strata, and BMI × age group interactions were not significant (endometrioma p = 0.453; dermoid p = 0.063; **Supplementary Table S6**; see Additional file). Using WHO BMI categories, age-adjusted geometric mean AMH showed no significant group × BMI-category interaction (p = 0.211), indicating similar categorical BMI patterns across groups (**Supplementary Table S7**; **Supplementary Figure S3**; see Additional file).

### Sensitivity analyses

Restricting to BMI ≤35 kg/m², the inverse BMI–AMH association persisted in both groups (dermoid −3.17% per kg/m², p=0.020; endometrioma −2.05%, p=0.044). In contrast, when restricting to BMI ≤30 kg/m², estimates attenuated and were not significant (dermoid −3.11%, p=0.084; endometrioma −1.16%, p=0.303), and group × BMI interactions remained non−significant across ranges (**Supplementary Table S8**; **Supplementary Figure S4**; see Additional file). This pattern is consistent with range restriction and a modestly non−linear BMI effect suggested by spline tests (**Supplementary Table S3**; see Additional file). Model diagnostics did not reveal major violations of assumptions (**Supplementary Figure S5**; see Additional file).

## Discussion

In this retrospective cohort of women with untreated ovarian endometrioma and dermoid cyst, higher BMI was associated with slightly lower AMH. However, the incremental explanatory contribution of BMI was small (partial R² = 0.01), and there was no statistical evidence that the BMI–AMH association differed by diagnosis (BMI×diagnosis interaction p = 0.538).In women without PCOS, many studies have reported that the association between BMI and AMH is generally weak or absent, although substantial heterogeneity exists across studies (13). For example, in the study by Albu et al. including 2,204 women, a weak positive correlation between BMI and AMH was observed when restricting the analysis to women with normal weight (14). By contrast, in the multicenter study by Jaswa et al., BMI was inversely associated with AMH overall, but the correlation was significant only among non-obese women (BMI <30 kg/m²) and disappeared in the obese subgroup (11). Similarly, in a study of 1,654 African American women, BMI showed an overall negative association with AMH, but the significance was largely driven by the extremely obese group (BMI ≥40 kg/m²) (15). Ethnic differences have also been reported: one study found that the negative BMI–AMH association was evident only in White women but absent in other racial groups (16).

In our study cohort, fewer than 10% of women had a BMI ≥30 kg/m², and cases of extreme obesity were uncommon. This may help explain our findings. Previous reports suggest that the negative BMI–AMH association becomes more evident only at very high BMI levels (e.g., ≥40 kg/m²). In contrast, the correlation may weaken or disappear in obese groups. Thus, the overall negative trend observed in our analysis may have been driven by a small number of women with particularly high BMI. Consistently, the sensitivity analysis restricted to BMI ≤30 kg/m² showed no significant association, aligning with patterns reported in non-PCOS cohorts. Nonetheless, given that the group (disease) × BMI interaction effect was not statistically significant, caution is warranted against overinterpreting differences in slopes between disease groups.

Clinically, these results highlight that BMI has limited utility as a surrogate for ovarian reserve in women with endometriosis. Using the fully adjusted estimate, a 5 kg/m² higher BMI corresponded to only 11% lower AMH, which is modest relative to age−related differences observed in the same model. Moreover, BMI explained only 1% of the remaining (residual) variance in AMH aftern covariate adjustment (partial R² = 0.01). This underscores that statistically significant associations may have limited value for individual−level prediction, and that BMI’s influence is minimal relative to other factors. These findings are consistent with prior literature describing generally weak and heterogeneous BMI–AMH associations in non−PCOS populations (11, 13).

These findings are consistent with the hypothesis that, in endometriosis (and specifically ovarian endometrioma), local ovarian microenvironmental factors (e.g., adipokines, inflammation, and angiogenesis) may contribute to AMH regulation beyond what is captured by systemic BMI. Importantly, because these pathways were not directly measured in our cohort, the following mechanistic discussion is hypothesis-generating and based on prior experimental and meta-analytic evidence. Leptin has been shown, in experiments using human granulosa cells, to suppress AMH mRNA expression via the JAK2/STAT3 pathway, whereas adiponectin has no such effect (17). In women with endometriosis, leptin concentrations are elevated in peritoneal and follicular fluid (18). However, serum levels remain inconsistent across studies (18), This raises the possibility that local leptin exposure may be elevated and not well reflected by BMI, even among women with similar BMI. Another meta-analysis showed that serum leptin concentrations and leptin-to-BMI ratios are significantly increased in women with endometriosis, whereas adiponectin levels are decreased (19). These findings suggest that BMI alone may not fully capture heterogeneity in adipokine-related signaling. In support of this, an obesity-induced mouse model has implicated leptin signaling in promoting the growth of endometriotic lesions (20). Together, these data support a plausible hypothesis that leptin-related signaling and other local inflammatory pathways may influence granulosa-cell AMH expression in endometriosis. If so, systemic BMI may track AMH only weakly. However, these data do not establish that leptin mediates the BMI–AMH association, and our study cannot evaluate these pathways directly. In our cohort, the apparent attenuation of the BMI–AMH association at lower BMI ranges (in spline and BMI-range sensitivity analyses) is exploratory. It should not be mechanistically attributed within this dataset. One speculative interpretation is that local adipokine exposure may vary independently of BMI. Because adipokines (e.g., leptin/adiponectin), insulin resistance indices, and fat distribution measures were not measured in this cohort, these mechanistic interpretations remain speculative and should be viewed as hypotheses for future studies.

In non-endometriosis contexts such as PCOS, insulin resistance and hyperinsulinemia affect granulosa cell development and FSH signaling (21). Consistently, decreases in AMH have been reported following metformin treatment (22). However, endometriosis is not primarily driven by insulin resistance, and studies conducted in Asian populations, where the BMI distribution is narrower than in Western cohorts, suggest that systemic metabolic factors may exert only minimal influence on AMH. Therefore, in women with endometriosis, local ovarian and peritoneal microenvironmental factors are likely to play a relatively greater role in regulating AMH than systemic metabolic disturbances.

It is well established that AMH declines with increasing age, although substantial variability exists within the same age group (7). In our study, age emerged as the strongest negative predictor of AMH. Segmented regression identified an earlier modeled age breakpoint in the endometrioma group than in the dermoid group. Because these data are cross-sectional and breakpoint estimation is model-based, this finding should not be interpreted as demonstrating accelerated follicular depletion. Instead, it should be viewed as hypothesis-generating and confirmed in longitudinal studies. From a clinical counseling perspective, these findings support proactive assessment and counseling in the late reproductive years (≥35 years).

The strengths of this study include the use of a single-center cohort with uniform AMH assay methodology and a homogenous ethnic population. By restricting the cohort to newly diagnosed patients prior to treatment, potential confounding could be more effectively controlled. A further strength is the direct comparison between women with ovarian endometrioma and those with dermoid cyst. Dermoid cyst serves as a pragmatic ovarian−cyst comparator that is not characterized by the chronic inflammatory endometriosis milieu (5), rather than a population−based healthy control group. Key covariates such as age and menstrual cycle characteristics were accounted for, and the full spectrum of BMI values was utilized.

Several limitations should also be acknowledged. An important limitation relates to cohort comparability. The dermoid cohort comprised surgically treated, pathologically confirmed cases, whereas endometriomas were identified clinically at presentation based on imaging findings. These differing clinical pathways may introduce selection differences (e.g., referral patterns, indications for surgery, or disease chronicity) and limit direct etiologic comparability between groups. In addition, cyst type itself may influence AMH independent of BMI, as suggested by prior preoperative comparisons reporting lower AMH in women with endometriomas than in age− and BMI−matched women with mature cystic teratomas (10). Although we adjusted for cyst size and laterality, residual confounding by disease phenotype cannot be excluded. Because this was a single-center, ethnically homogeneous cohort with a relatively narrow BMI distribution and few participants with BMI ≥30 kg/m², generalizability to multi-ethnic and Western populations with wider BMI spectra and higher obesity prevalence may be limited. In addition, BMI does not capture body composition, fat distribution, or metabolic biomarkers (e.g., leptin/adiponectin, insulin resistance indices), which were not available in this dataset. Finally, the cross-sectional design precludes causal inference.

Future studies incorporating refined phenotyping, including not only serum but also follicular fluid and peritoneal fluid profiles of adipokines, inflammatory mediators, and iron metabolism, will be necessary to better elucidate the relationship between “metabolic exposures” beyond BMI and AMH.

## Conclusion

In conclusion, our findings indicate that BMI shows a modest negative association with AMH in women with endometrioma. Given BMI’s minimal incremental explanatory value beyond covariates, this relationship appears to be attenuated at lower BMI ranges and not significantly different from that observed in comparator. Taken together with prior literature, these results suggest that systemic BMI may not fully capture the metabolic influences on ovarian reserve in endometrioma. Local microenvironmental factors (e.g., inflammatory and adipokine-related pathways) may play an important role, but this requires direct evaluation in studies with integrated metabolic profiling. Further studies with broader BMI distributions and integrated metabolic profiling are warranted to clarify the complex interplay between adiposity and ovarian reserve in this population.

## Data Availability

De-identified data supporting the findings of this study are available from the corresponding author upon reasonable request.
