# Supplementary material for: Association between body mass index and anti-Müllerian hormone in women with ovarian endometrioma and dermoid cyst
Source: Front Endocrinol (Lausanne). 2026 Feb 19;17:1746451. doi: 10.3389/fendo.2026.1746451 (PMC12961198; doi:10.3389/fendo.2026.1746451)

**Table S1.** Age-adjusted comparison of geometric mean AMH between the two groups. ANCOVA with age as a covariate was performed to estimate the geometric mean AMH values in the endometrioma (Endo) and dermoid cyst groups (Derm). Estimates were calculated on the log scale and then back-transformed to the original unit (ng/mL), with 95% confidence intervals (CIs). The ratio of AMH between groups with its 95% CI is also presented, and the p-value corresponds to the significance of the difference in AMH between the two groups.

| Group | Adjusted geometric mean AMH | 95% CI | p-value |
| --- | --- | --- | --- |
| Endometrioma | 2.18 | 2.05-2.31 |  |
| Dermoid | 2.34 | 2.10-2.60 |  |
| Ratio Endo/Derm | 0.93 | 0.82-1.05 | 0.245 |
| Ratio Derm/Endo | 1.08 | 0.95-1.22 | 0.245 |

**Table S2**. Segmented regression analysis of age-related changes in AMH in women with endometrioma and dermoid cyst.

For each group, a breakpoint was estimated in the age–AMH relationship, occurring at 35.7 years in the endometrioma group and at 40.4 years in the dermoid cyst group. The table presents the estimated slopes (β ± standard error) of log-transformed AMH with age before and after the breakpoint, along with p-values for between-group differences in slopes within each segment.

| Comparison | Breakpoint (years) | Endometrioma (β ± SE) | Dermoid (β ± SE) | p-value |
| --- | --- | --- | --- | --- |
| Before breakpoint | 35.7 (Endo), 40.4 (Derm) | –0.03 ± 0.01 | –0.03 ± 0.01 | 0.790 |
| After breakpoint | 35.7 (Endo), 40.4 (Derm) | –0.21 ± 0.02 | –0.51 ± 0.15 | 0.047 |

**Table S3**. Comparison of age-adjusted linear and restricted cubic spline (RCS, df = 4) models for the BMI–AMH relationship.

Log-transformed AMH was regressed on BMI with age adjustment. Model fit was compared between a linear specification and an RCS specification. For each model, the residual degrees of freedom (df) and residual sum of squares (RSS) are shown, together with the difference in df (Δdf), difference in sum of squares (ΔSS), F statistic, and p-value. The RCS model provided a significantly better fit than the linear model.

| Model | Residual df | RSS | Δdf | ΔSS | F | p-value |
| --- | --- | --- | --- | --- | --- | --- |
| RCS (df=4) | 942 | 566.49 | Ref | Ref | Ref | Ref |
| Linear | 945 | 573.86 | 3 | –7.37 | 4.08 | 0.007 |

**Table S4**. Association between BMI and serum AMH in women with endometrioma and dermoid cysts across sequential multivariable regression models.

Log-transformed AMH was modeled using restricted cubic spline functions (df = 4). An interaction term (BMI × Group) was included in all models to test whether the BMI–AMH association differed between groups. Additional covariates were introduced stepwise:

Model 1: Age + Group × BMI

Model 2: Model 1 + Cycle regularity

Model 3: Model 2 + Smoking

Model 4: Model 3 + Cyst maximum diameter

Model 5: Model 4 + Parity

Results are expressed as percent change in AMH per 1 kg/m² increase in BMI (95% CI) and as the corresponding absolute change in AMH (ng/mL) after back-transformation. Interaction rows indicate the differential BMI effect between groups.

| Model | Group | % change per 1 BMI (95% CI) | Absolute change per 1 BMI (95% CI, ng/mL) | *p*-value |
| --- | --- | --- | --- | --- |
| Model 1 | Endometrioma | −1.9 % (−3.8 to −0.0) | −0.06 (−0.11 to 0.00) | 0.047 |
|  | Dermoid | −2.8 % (−5.1 to −0.5) | −0.10 (−0.17 to −0.03) | 0.020 |
|  | Interaction | +0.9 % (−2.2 to 4.0) | +0.05 (−0.04 to 0.14) | 0.573 |
| Model 2 | Endometrioma | −1.7 % (−3.6 to 0.3) | −0.06 (−0.11 to 0.00) | 0.092 |
|  | Dermoid | −2.8 % (−5.1 to −0.4) | −0.11 (−0.18 to −0.03) | 0.023 |
|  | Interaction | +1.2 % (−1.9 to 4.4) | +0.05 (−0.04 to 0.14) | 0.468 |
| Model 3 | Endometrioma | −1.5 % (−3.5 to 0.4) | −0.05 (−0.11 to 0.00) | 0.119 |
|  | Dermoid | −2.7 % (−5.0 to −0.3) | −0.10 (−0.17 to −0.03) | 0.031 |
|  | Interaction | +1.2 % (−2.0 to 4.4) | +0.04 (−0.05 to 0.14) | 0.471 |
| Model 4 | Endometrioma | −1.4 % (−3.3 to 0.5) | −0.05 (−0.11 to 0.01) | 0.154 |
|  | Dermoid | −2.5 % (−4.9 to −0.1) | −0.09 (−0.16 to −0.02) | 0.043 |
|  | Interaction | +1.1 % (−2.0 to 4.3) | +0.04 (−0.05 to 0.13) | 0.483 |
| Model 5 | Endometrioma | −1.5 % (−3.4 to 0.5) | −0.05 (−0.11 to 0.01) | 0.135 |
|  | Dermoid | −2.5 % (−4.8 to −0.0) | −0.09 (−0.16 to −0.02) | 0.048 |
|  | Interaction | +1.0 % (−2.1 to 4.2) | +0.04 (−0.05 to 0.13) | 0.538 |

Overall model fit was modest (adjusted R² = 0.22); see Table S5 for detailed model fit indices.

**Table S5**. Model fit indices for sequential multivariable models examining the association between BMI and log-transformed AMH.

Each model was compared to the preceding one to assess incremental explanatory value (ΔR², ΔAIC). Adjusted R² remained approximately 0.22 across models, and the partial R² for BMI was = 0.01, indicating that BMI explained 1% of the total variance in AMH.

| Model | Covariates added | R² | Adjusted R² | AIC | ΔAIC (vs Model 1) | Partial R² for BMI |
| --- | --- | --- | --- | --- | --- | --- |
| 1 | Age + Group × BMI | 0.23 | 0.22 | 1428.6 | – | 0.012 |
| 2 | + Cycle regularity | 0.23 | 0.22 | 1429.2 | +0.6 | 0.012 |
| 3 | + Smoking | 0.23 | 0.22 | 1429.4 | +0.8 | 0.011 |
| 4 | + Cyst max diameter | 0.23 | 0.22 | 1429.5 | +0.9 | 0.011 |
| 5 | + Parity | 0.24 | 0.22 | 1429.1 | +0.5 | 0.012 |

Partial R² for BMI denotes the incremental proportion of variance in log‑AMH explained by adding BMI to the multivariable model containing the other covariates.

**Table S6**. Age-stratified correlation between BMI and AMH and interaction tests by age cutoffs.

Pearson’s correlation coefficients (r) with p-values and sample sizes (n) are shown for each subgroup defined by the estimated breakpoint. Interaction coefficients and p-values are derived from linear regression models of log-transformed AMH including BMI, age group (≤ vs > cutoff), and the BMI × age group interaction, with age modeled using restricted cubic splines (df = 4).

| Group | Age cutoff | ≤ cutoff (r, p, n) | > cutoff (r, p, n) | Interaction coefficient | p (BMI × age group) |
| --- | --- | --- | --- | --- | --- |
| Endometrioma | 35.7 | r = –0.08, p = 0.088, n = 522 | r = –0.09, p = 0.200, n = 194 | –0.02 | 0.453 |
| Dermoid | 40.4 | r = –0.07, p = 0.285, n = 219 | r = –0.33, p = 0.252, n = 14 | –0.11 | 0.063 |

**Table S7**. Age-adjusted geometric mean AMH across BMI categories (WHO classification) within each group.

Models of log-transformed AMH were fitted with BMI category and age (restricted cubic spline, df = 4), separately within the endometrioma and dermoid cyst groups. Values are geometric means with 95% confidence intervals (CIs), back-transformed from the log scale. p-values are from comparisons with the Normal category using Dunnett adjustment. The rightmost column presents the ratio of dermoid versus endometriosis within each BMI category (95% CI). The Group × BMI category interaction was tested using an omnibus F-test.

| BMI category | Endometrioma (geo mean, 95% CI) | p (vs Normal) | Dermoid (geo mean, 95% CI) | p (vs Normal) | Ratio Dermoid / Endometrioma (95% CI) | p-value |
| --- | --- | --- | --- | --- | --- | --- |
| Underweight (<18.5) | 3.08 (2.58–3.68) | 0.046 | 2.50 (1.79–3.49) | 0.611 | 0.87 (0.61–1.24) | 0.429 |
| Normal (18.5–24.9) | 2.56 (2.26–2.90) | Ref | 2.85 (2.37–3.42) | Ref | 1.18 (1.03–1.35) | 0.019 |
| Overweight/Obese ≥25 | 2.35 (1.87–2.95) | 0.650 | 2.21 (1.73–2.83) | 0.581 | 0.99 (0.73–1.35) | 0.967 |
| Interaction (BMI cat x Group) |  |  |  |  |  | 0.211* |

*p(Group × BMI category interaction, omnibus F-test)

**Table S8**. Sensitivity analyses of the BMI–AMH association under alternative BMI inclusion ranges.

Log-AMH was regressed on BMI (per 1 kg/m²) with age adjusted via restricted cubic splines (df=4), and an interaction term (BMI × Group) included in all models. Results are shown as percent change (95% CI) and absolute change (ng/mL, 95% CI) per 1 kg/m², with two-sided p-values. Panel (a) shows group-level distribution of higher BMI; Panel (b) presents regression results for BMI ≤30, and ≤35 kg/m². Interaction terms were non-significant across all ranges.

(a) Group distribution of higher BMI

| Group | Total (n) | BMI ≥25 | BMI ≥30 | BMI ≥35 |
| --- | --- | --- | --- | --- |
| Endometrioma | 716 | 63 (8.8%) | 11 (1.5%) | 2 (0.3%) |
| Dermoid | 234 | 43 (18.4%) | 14 (6.0%) | 3 (1.3%) |

(b) Regression results by BMI range

| BMI range | Group | % change per 1 BMI (95% CI) | Absolute change (95% CI, ng/mL) | p-value |
| --- | --- | --- | --- | --- |
| ≤30 kg/m² (a) | Dermoid | –3.11% (–6.64 to 0.42) | –0.11 (–0.29 to 0.08) | 0.084 |
|  | Endometrioma | –1.16% (–3.36 to 1.05) | –0.033 (–0.17 to 0.10) | 0.303 |
|  | Interaction | 1.97% (–2.17 to 6.29) | — | 0.356 |
| ≤35 kg/m² (b) | Dermoid | –3.17% (–5.84 to –0.49) | –0.11 (–0.29 to 0.08) | 0.020 |
|  | Endometrioma | –2.05% (–4.05 to –0.06) | –0.06 (–0.19 to 0.08) | 0.044 |
|  | Interaction | 1.12% (–2.20 to 4.55) | — | 0.512 |

**Figure S1**. Flow chart

**Figure S2**. Age versus AMH levels in women with endometrioma and dermoid cyst. Satter plots of age against AMH (ng/mL) for both groups, overlaid with locally weighted regression (LOWESS) curves to illustrate overall distribution and trends.


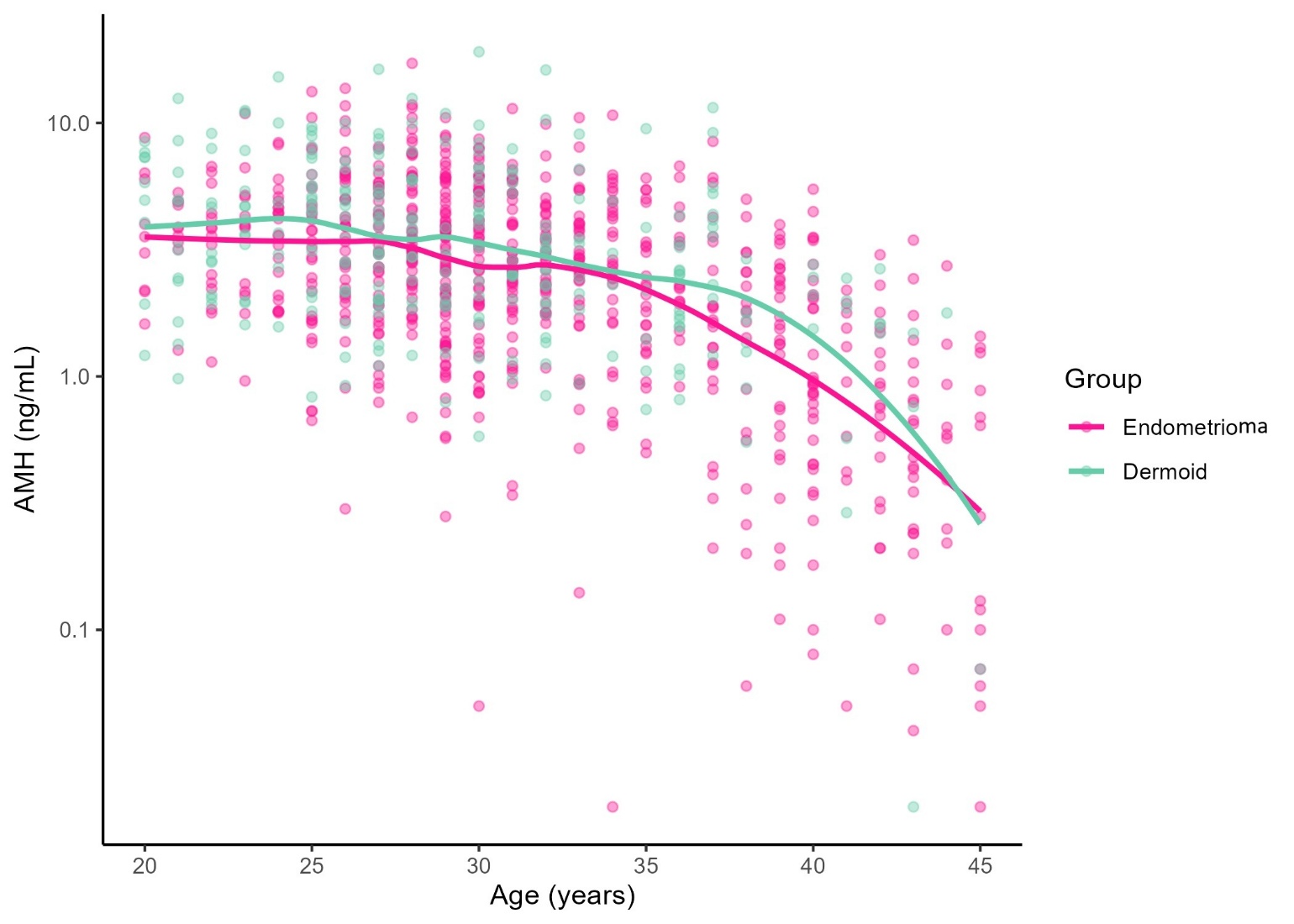


**Figure S3**. Age-adjusted geometric mean AMH across BMI categories (WHO classification). Points represent estimated geometric means (back-transformed from the log scale), with error bars indicating 95% confidence intervals. Results are shown separately for endometrioma (pink) and dermoid cysts (mint).


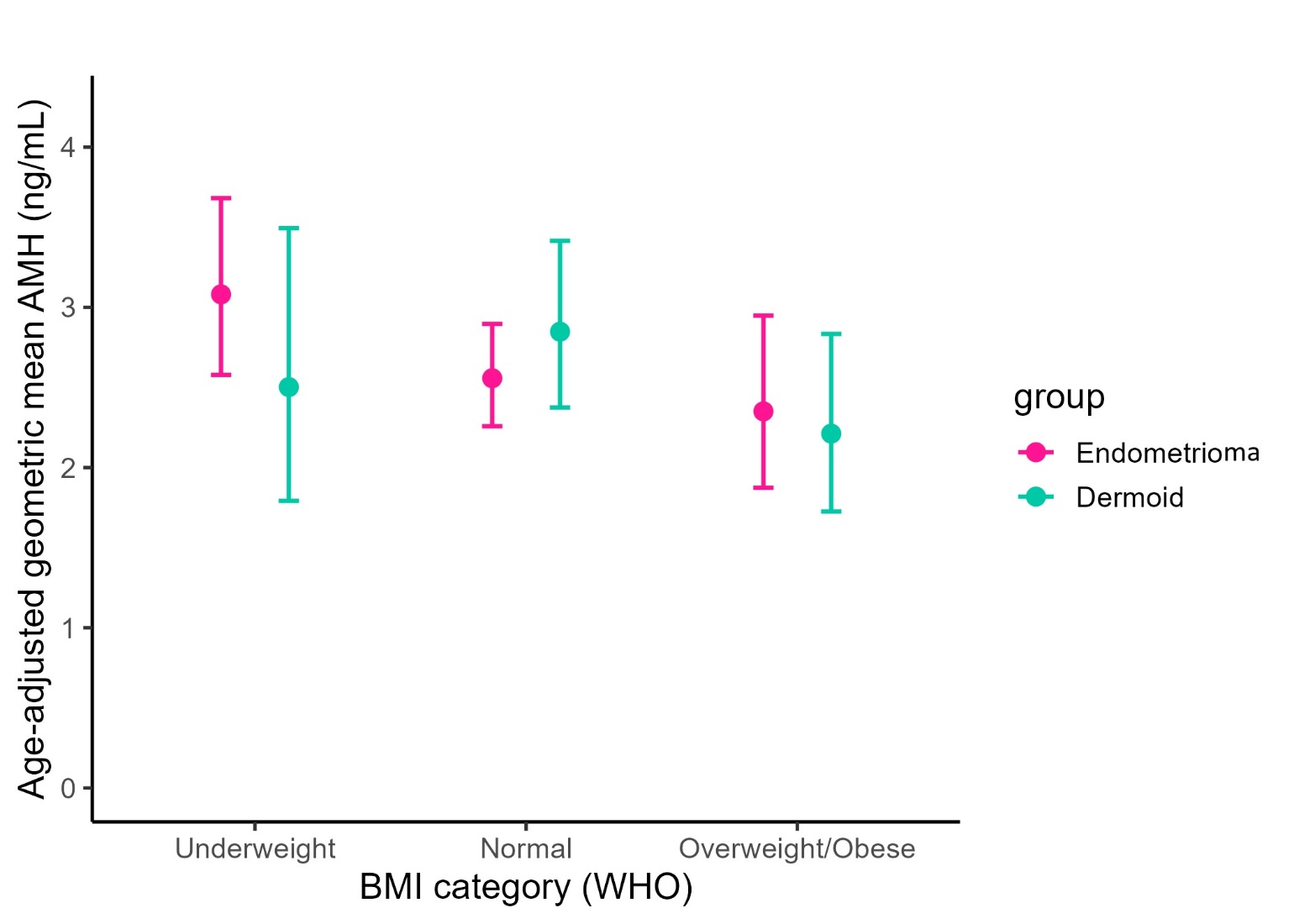


**Figure S4**. Distribution of BMI and AMH across study groups.

Combined scatterplot showing individual data points, with color indicating group (endometrioma in pink, dermoid in mint) and point shapes indicating BMI cutoffs (<25, 25–29.9, 30–34.9, ≥35 kg/m²). The y-axis is displayed on a log scale to accommodate the skewed distribution of AMH.


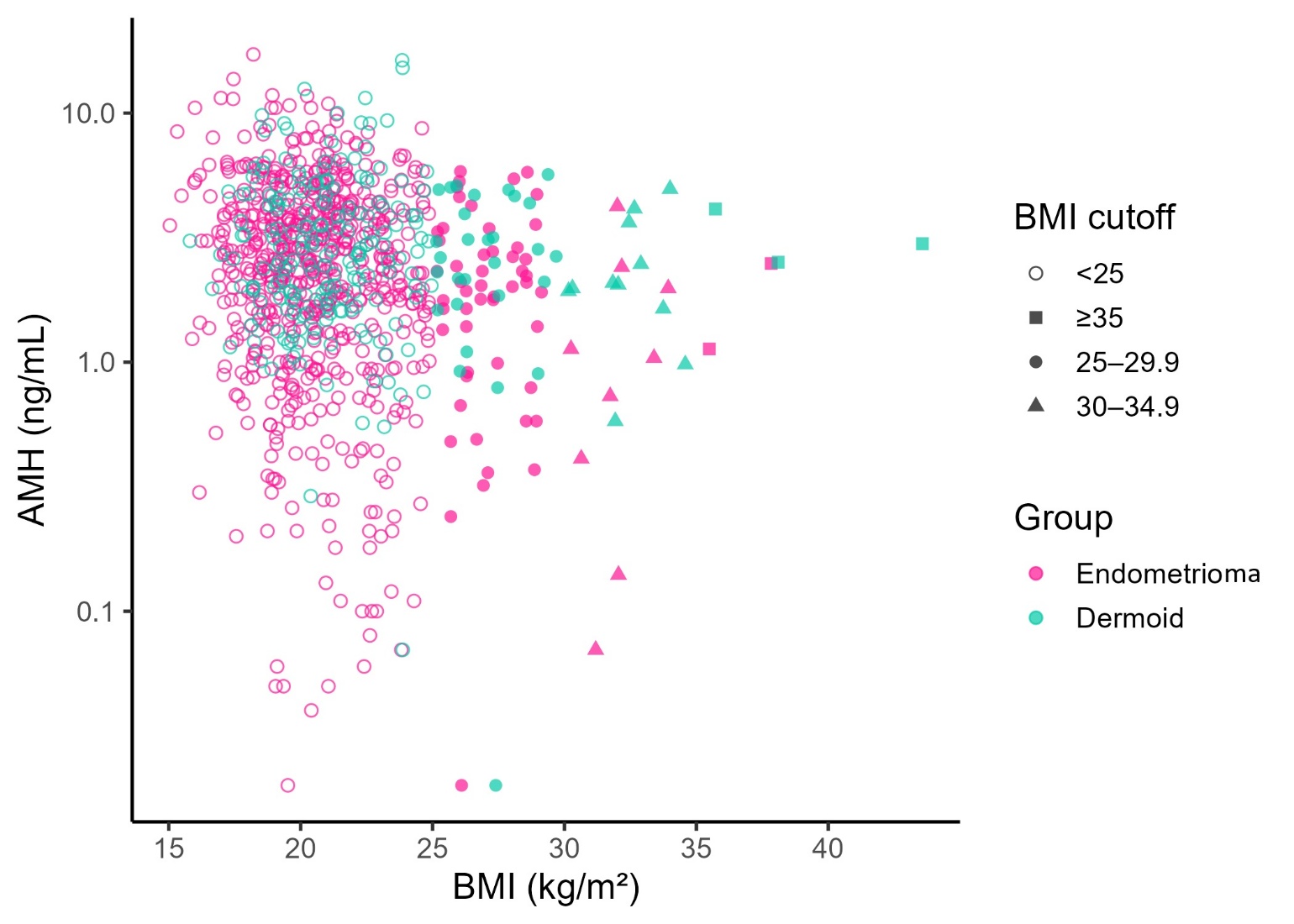


**Figure S5**. Diagnostic plots of regression models with restricted cubic splines (RCS) for women with endometrioma and dermoid cysts. For each model, four standard diagnostic plots are presented: (A) residuals versus fitted values, (B) normal Q–Q plot, (C) scale–location plot, and (D) residuals versus leverage. Visual inspection revealed no major violations of model assumptions.


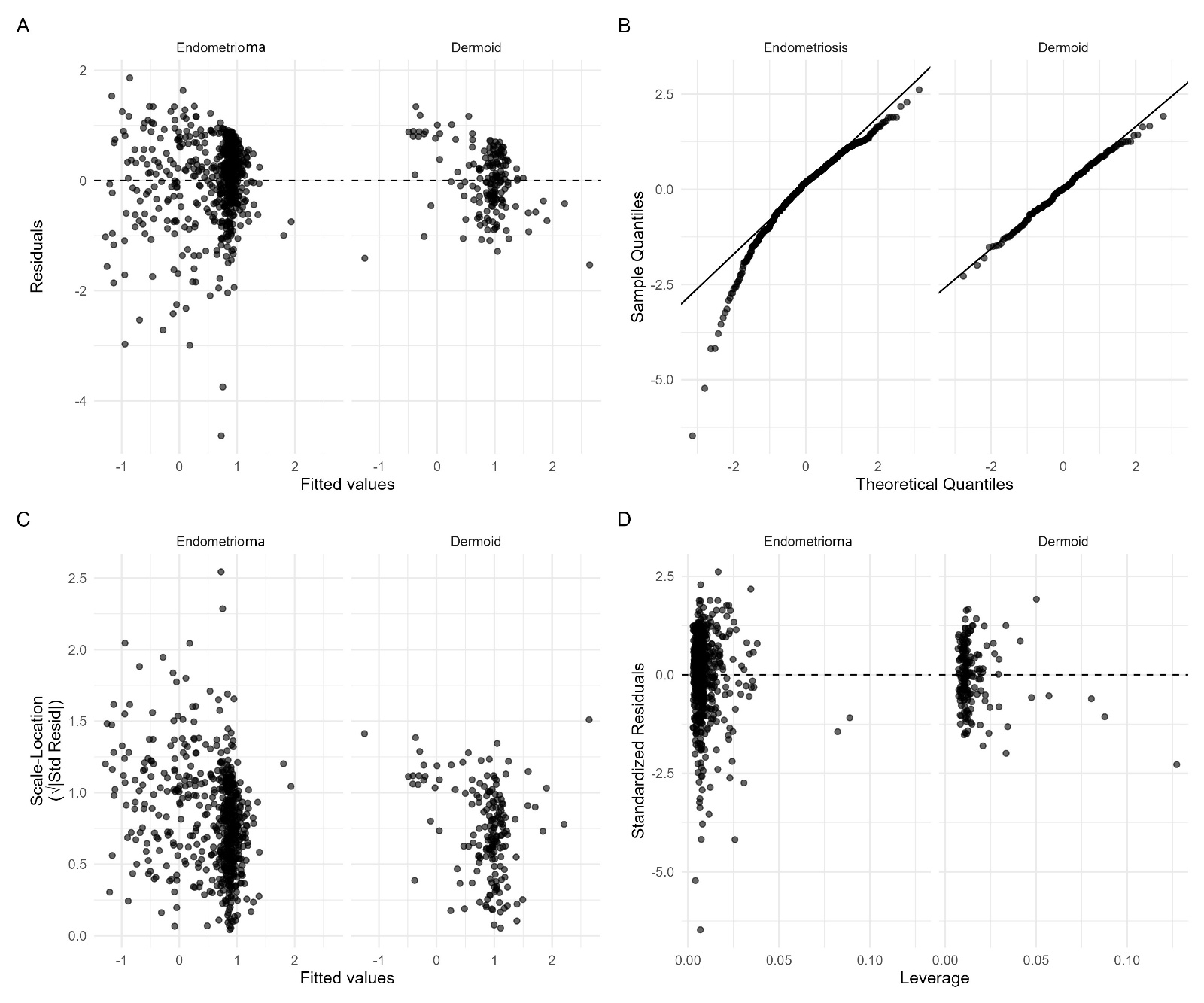

Supplement: Supplementary file 1 [file DataSheet1.docx]
